# Adverse events in children and adolescents undergoing allergen immunotherapy for respiratory allergies—Report from the Allergen Immunotherapy Adverse Events Registry (ADER), a European Academy of Allergy and Clinical Immunology taskforce

**DOI:** 10.1002/clt2.12250

**Published:** 2023-06-02

**Authors:** Julijana Asllani, Dimitrios Mitsias, George Konstantinou, Eris Mesonjesi, Fatmira Xhixha, Esmeralda Shehu, George Christoff, Katia Noleva, Michael Makris, Xenofon Aggelidis, Mirjana Turkalj, Erceg Damir, Ioana Agache, Vesna Tomic‐Spiric, Rajica Stosovic, Zeynep Misirligil, Mitja Kosnik, Todor A. Popov, Moises Calderon, Nikolaos G. Papadopoulos, ADER study group, Gerta Sinani, Eris Mesonjesi, Esmeralda Shehu, Etleva Qirko, Anila Nano, Valbona Martini, Enkelejda Gjata, Aferdita Sinani, Jana Sinani, Sybi Musollari, Alketa Bakiri, Diana Qama, Erjola Piluri, Fatmira Xhixha, Gilda Xhoxhi, Mirela Hitaj, Mehmet Hoxha, Alfred Priftanji, Dimitrov Zlatko, George Christoff, Silviya Novakova, Katya Noleva, Plamen Yakovliev, Ted Popov, Mirjana Turkalj, Damir Erceg, Asja Stipic, Mira Pevec, Branko Pevec, Sanja Popovic‐Grle, Nikos Papadopoulos, Michael Makris, George Konstantinou, Dimitrios Mitsias, Xenofon Aggelidis, Christina Ciobanum, Ioana Agache, Florin Dan Popescu, Irina Bukur, Ierima Augustin, Deleanu Dianna, Adriana Muntean, Vesna Tomic‐Spiric, Rajica Stosovic, Mirjana Bogic, Dragana Tadic, Aleksandra Plavsic, Mitja Kosnik, Zeynep Mısırlıgil, Dilsad Mungan, Moises A. Z. Calderon

**Affiliations:** ^1^ University of Medicine Tirana Albania; ^2^ Allergy and Asthma Medical Clinic Tirana Albania; ^3^ Allergy Department 2^nd^ Pediatric Clinic University of Athens Athens Greece; ^4^ Department of Allergy and Clinical Immunology 424 General Military Training Hospital Thessaloniki Greece; ^5^ University Hospital Center “Mother Teresa” Tirana Albania; ^6^ Ambulatory Polyclinic of Specialties, Nr. 3 Tirana Albania; ^7^ Internal Medicine Department Durres Regional Hospital Durres Albania; ^8^ Medical University‐Sofia Faculty of Public Health Sofia Bulgaria; ^9^ ET Dr. K. Noleva IPSMP Sofia Bulgaria; ^10^ Immunotherapy Outpatient Clinic Allergy Unit 2^nd^ Department Dermatology and Venereology National and Kapodistrian University of Athens University General Hospital ‘Attikon’ Athens Greece; ^11^ Srebrnjak Children's Hospital Zagreb Croatia; ^12^ Medical School Catholic University of Croatia Zagreb Croatia; ^13^ Medical School University J.J. Strossmayer Osijek Croatia; ^14^ Faculty of Medicine Transylvania University Brasov Romania; ^15^ Clinic of Allergology and Immunology University Clinical Center of Serbia Belgrade Serbia; ^16^ Faculty of Medicine University of Belgrade Belgrade Serbia; ^17^ İstinye University Faculty of Medicine Ankara Liv Hospital Ankara Turkey; ^18^ University Clinic of Respiratory and Allergic Diseases Golnik Slovenia; ^19^ University Hospital "Sv Ivan Rilski" Sofia Bulgaria; ^20^ Section of Allergy and Clinical Immunology Imperial College London‐NHLI London UK; ^21^ Lydia Becker Institute of Immunology and Inflammation University of Manchester Manchester UK

**Keywords:** adverse events, allergen immunotherapy, real‐life settings, risk factors

## Abstract

**Background:**

Although it has been shown that allergen immunotherapy (AIT) is well‐tolerated in children, systematic and prospective surveillance of AIT safety in real life settings is needed.

**Methods:**

The multinational Allergen Immunotherapy Adverse Events Registry (ADER) was designed to address AIT safety in real life clinical practice. Data on children ≤18 years old with respiratory allergies undergoing AIT were retrieved. Patient‐ and AIT‐related features were collected and analyzed. The characteristics of adverse events (AE) and risk factors were evaluated.

**Results:**

A total of 851 patients, 11.3 ± 3.4 years old, with rhinitis only (47.6%); asthma and rhinitis (44.5%); asthma (7.9%), receiving 998 AIT courses were analyzed. Sublingual immunotherapy (SLIT) accounted for 51% of the courses. In 84.5% of patients only one AIT treatment was prescribed. Pollen was the most frequent sensitizer (57.1%), followed by mites (53.4%), molds (18.2%) and epithelia (16.7%). Local and systemic AEs were reported in 85 patients (9.9%). Most AEs (83.1%) were mild and occurred in <30 min (87%). Respiratory and cutaneous symptoms were more frequent. Only 4 patients (0.47%) had severe AE (none after 6 weeks of maintenance). The risk of AE was higher in patients undergoing SCIT.

**Conclusions:**

AIT is safe and well tolerated in children and adolescents with respiratory allergies in real‐life clinical practice. Though SCIT is more prone to AE compared to SLIT, overall severe reactions are rare and occur during build‐up and early maintenance.

## 1 INTRODUCTION

Allergen immunotherapy (AIT) is currently the only disease modifying treatment option for children and adults with IgE‐mediated allergic disorders. Both subcutaneous (SCIT) and sublingual (SLIT) routes are proven to be effective in providing short‐ and long‐term benefits in allergic patients that may persist after discontinuation.^1^, ^2^, ^3^ It has been shown that allergen immunotherapy in children is well‐tolerated, but there are still concerns regarding safety.^4^, ^5^, ^6^, ^7^, ^8^ Even though few observational studies and RCTs have evaluated adverse events (AE) due to AIT, there is a need for systematically and prospectively documenting these AE, in the context of an AIT registry.^9^, ^10^, ^11^, ^12^, ^13^ Such a registry (Allergen Immunotherapy Adverse Reactions Registry ‐ ADER) has been established with the support of the European Academy of Allergy and Clinical Immunology.^14^ Moreover, it is important to identify and highlight factors that may increase the risk of AE. Although several previous reports have assessed several risk factors associated with AE during AIT, data regarding the pediatric population are lacking.^9^, ^15^, ^16^, ^17^, ^18^ In this report, we describe the characteristics and evaluate risk factors for adverse reactions in children and adolescents with allergic rhinoconjunctivitis and/or asthma who underwent AIT for pollen, mites, molds and/or epithelia.

## 2 METHODOLOGY

The overall methodology of the ADER registry has been described.^14^ In short, ADER is a prospective, observational, multicenter, web‐based registry of AIT in real‐life clinical practice conducted in eight South Eastern‐European countries: Albania (AL), Bulgaria (BG), Croatia (HR), Greece (GR), Romania (RO), Serbia (RS), Slovenia (Sl) and Turkey (TR). The data were collected through three questionnaires developed by the ADER Task Force (additional questions were added to assess local reactions).^14^ AEs were recorded according to the Medical Dictionary for Regulatory Activities (MedDRA) terminology.^19^ The Research Electronic Data Capture (REDCap) electronic platform was used to ensure safe uploading and storage of the registry.^20^

### 2.1 Ethics

ADER complies with national and European ethical and regulatory requirements, including data protection. Each NC was responsible for obtaining study approval from its corresponding national or local independent Ethics Committee.

### 2.2 Study population and adverse event recording

Among subjects registered in ADER, we have retrieved in this study children and adolescents ≤18 years old with allergic rhinitis and/or conjunctivitis and/or asthma who underwent SCIT or SLIT for mites, pollens, molds (alternaria) and/or epithelia. A total of 851 patients receiving 998 courses of AIT were included. They were 11.3 ± 3.4 years old and most of them were males (63.2%). There were 480 (56.4%) children (*n* = 52 preschool, aged ≤6 years old) and 371 (43.6%) adolescents. Data on sensitization, past medical history and allergy history, current treatments, and characteristics of the current AIT course(s) were collected from the patient's clinical files. Patients suffered from allergic rhinitis (AR) alone in 47.6% of cases, AR and asthma in 44.5% and asthma only in 7.9%. Poly‐sensitization was observed in 53% of patients. Pollens were the most frequent sensitizer in 57.1%, followed by mites (53.4%), molds (18.2%) and epithelia (16.7%). SLIT accounted for 51% of all treatments [*n* = 506 courses (drops in 485; tablets in 20; 1 course unspecified)], prescribed in 446 (52.5%) patients, while SCIT was prescribed in 405 patients (492 courses). In the majority of cases (84.4%) only one AIT course was administered, two treatment courses in 14.2% and more than two in only 12 patients (Table 1). Non‐allergic comorbidities were also recorded but were rare: gastrointestinal disease in 2 cases, dermatologic in 1 case, psychiatric in 3 and rheumatic and pulmonary conditions in 1 case each. Both systemic and local AE that occurred during AIT were recorded in detail. The severity of AE was classified (a) according to the Muller grade classification system,^21^ and (b) as mild, moderate and severe.^22^ We also recorded the phase when the AEs occurred (build‐up or maintenance), treatment, the time of onset and resolution, final outcomes and possible modification of the treatment course. Systemic adverse reactions were recorded using MeDRA.^19^ Local reactions related to SCIT or SLIT were collected and classified as (a) local skin symptoms, (b) large local cutaneous reactions, (c) local mouth symptoms, and (d) oral edema.

**TABLE 1:** Characteristics of the patients with an adverse event compared with those without.

	Whole population *n*	Without AE *n* (%)	With AE *n* (%)
Patients	851	766 (90)	85 (9.9)
Age groups (3–11 years old)	480 (56.4)	435 (90.6)	45 (9.4)
12–18 years old	371 (43.6)	331 (89.2)	40 (10.8)
Sex (Male)	538 (63.2)	487 (90.5)	51 (9.5)
Female	313 (36.8)	279 (89.1)	34 (10.9)
Age (mean ± SD) years	11.3 ± 3.5	11.3 ± 3.5	11.9 ± 3.4
Countries			
Albania	269 (31.6)	248 (92.2)	21 (7.8)
Bulgaria	250 (29.4)	236 (94.4)	14 (5.6)
Greece	280 (32.9)	252 (90)	28 (10)
Croatia	15 (1.8)	3 (20)	12 (80)
Romania	20 (2.4)	14 (70)	6 (30)
Serbia	13 (1.5)	11 (84.6)	2 (15.4)
Allergic condition			
AR	405 (47.6)	360 (88.9)	45 (11.1)
AR and asthma	379 (44.5)	344 (90.8)	35 (9.2)
Asthma	67 (7.9)	62 (92.5)	5 (7.5)
Comorbidities			
Atopic dermatitis	72 (9.1)	64 (88.9)	8 (11.1)
Food allergy	33 (3.9)	27 (81.8)	6 (18.2)
Chronic urticarial	6 (0.7)	4 (66.7)	2 (33.3)
Drug allergy	7 (0.8)	7 (100)	0 (0)
Mono‐sensitized	401 (47.1)	364 (90.8)	37 (9.2)
Poly‐sensitized	450 (53)	402 (89.4)	48 (10.6)
Previous anaphylaxis	6	6 (100)	0 (0)
Number of SIT courses			
1	718 (84.4)	649 (90.4)	69 (9.6)
2	121 (14.2)	108 (89.3)	13 (10.7)
More than 2 courses	12 (1.4)	9 (75)	3 (25)
Single‐allergen AIT	771 (90.6)	697 (90.4)	74 (9.6)
Mixtures	80 (9.4)	70 (87.5)	10 (12.5)

### 2.3 Statistical analysis

Means and standard deviations were used to evaluate normally distributed continuous data and frequencies and percentages for categorical data. Chi square test or Fisher's Exact test were used to assess correlations between categorical variables. Non‐normally distributed variables were analyzed with non‐parametric U Mann–Whitney test. Logistic regression was used to evaluate possible risk factors among patients who reported AE (dependent variable). Age, sex, allergic disease, comorbidities, route of administration, allergen sensitization profile, current AIT treatments, the number of treatments and previous AIT treatments were included as possible risk factors. Data were analyzed using SPSS software (version 26.00 IBM) for Windows and a probability value of less than 0.05 was considered statistically significant.

## 3 RESULTS

### 3.1 Characteristics of patients with AE compared with those without

A total of 250 AEs were reported in 85/851 patients (9.9%) with a mean age of 11.9 ± 3.4 years. There were no significant differences between patients with and without AE regarding patient‐related factors such as age, sex, allergic conditions, comorbidities, mono or polysensitization and number of AIT courses. Among patients with AE, 60% (*n* = 51) were males, the majority (53%) had allergic rhinitis only and 56.5% (*n* = 48) were polysensitized. Most were receiving AIT with mites (43.5%) followed by AIT with grass pollen extract in 35.3% (*n* = 30). Atopic dermatitis was present in 9.4% (*n* = 8) of the cases with AE and none of them had reported a previous history of anaphylaxis. The proportions of patients with AE differed significantly between countries, ranging from 80% in HR to 30% in RO, 15.4% in RS, 10% in GR, 7.8% in Al and 5.6% in BG. Single allergen treatments were administered in most of the patients (90.6%), while mixtures were prescribed in only 9.4%. A slightly higher proportion of patients with AE were receiving mixtures (12.5%) rather than single allergens (9.6%) (Table 1).

A significantly higher proportion of patients receiving SCIT (*n* = 63; 15.5%) reported AE compared to those undergoing SLIT (*n* = 22; 4.9%) (*p* < 0.001). These proportions were also higher for specific allergens among patients undergoing SCIT: epithelia (27.3%) and grass pollen (22.7%) were associated with a higher proportion of patients with AE compared to other allergens: mites (13.4%), molds (6.5%) (Table 2). No significant difference was reported in the proportion of patients with AE receiving SCIT with allergoids (14.3%) versus natural extracts (15.6%). AEs were observed in 23.7% of those receiving SCIT with tyrosine followed by 15.6% for aluminum hydroxide and 10.8% with calcium phosphate (Table 2). For patients undergoing SLIT, AE did not differ significantly according to allergens. Similarly, SLIT formulation did not affect AE frequency: AE with SLIT drops (4.9%) and tablets (5%) were similar (Table 2). More than one reaction has been recorded in some patients (up to 10 reactions in one patient).

**TABLE 2:** Characteristics of the patients with adverse reactions according to the route of administration.

	Whole population *n* (%)		With AE *n* (%)	
	SCIT	SLIT	SCIT	SLIT
Patients	405	446	63 (*15.5)*	22 (*4.9)*
Sensitization profile (positive SPT/sIgE)				
Pollen	187 (46.1)	300 (67.3)	32 (17.1)	17 (5.7)
Mites	255 (62.9)	196 (43.9)	34 (13.3)	9 (4.6)
Epithelia	68 (16.8)	74 (16.4)	15 (22.1)	6 (8.1)
Molds	73 (18.1)	83 (18.4)	7 (9.6)	4 (4.8)
Current AIT allergen				
Pollen total	143 (35)	306 (67.2)	34 (23.7)	17 (5.5)
Grass*	79 (19.5)	194 (43.5)	18 (22.7)	12 (6.2)
Mites	239 (59)	162 (36.1)	32 (13.4)	5 (3.1)
Epithelia *	11 (2.7)	10 (2.2)	3 (27.3)	1 (10)
Molds	46 (11.3)	29 (6.5)	3 (6.5)	1 (3.4)
Type of extract				
Allergoid	21 (5.2)	na	3 (14.3)	na
Natural	384 (94.8)	446 (100)	60 (15.6)	22 (4.8)
Adjuvant^a^				
Aluminum hydroxide	244 (60.2)	na	38 (15.6)	na
Calcium phosphate	93 (23)	na	10 (10.8)	na
MPL	4 (1)	na	0	na
Tyrosine	38 (9.4)	na	9 (23.7)	na
Sublingual AIT formulations^a^				
Drops	na	425 (95.3)	na	21 (4.9)
Tablets	na	20 (4.5)	na	1 (5)
Previous AIT course	25	25 (6.1)	19 (4.1)	6 (24)

*Note*: The proportions of patients with AE by each variable according to route.

Abbreviations: AIT, allergen immunotherapy; MPL, Monophosphoryl Lipid A; na, not applicable.

^a^ missing cases.

*(*p* < 0.05).

Overall, the most frequently reported symptoms in patients with systemic AE were respiratory (cough 48%; rhinitis 43%; dyspnea 11%; chest tightness 9%) and skin (localized urticaria 10%; generalized urticaria 7%) (Supplementary Table S1).

### 3.2 Severity and treatment of adverse events

Ninety percent of the reactions (*n* = 225) occurred during SCIT, compared to 10% (*n* = 25) during SLIT (*p* < 0.001).

Among AEs, 89 local reactions were recorded (35.6%). In SCIT, there were 50 (22%) mild cutaneous and 25 (11%) large local reactions, while in SLIT, mild oral symptoms were recorded in 9 (36%) cases and oral edema in 5 cases (20%).

Most reactions were mild (83.1%, *n* = 108, recorded in 66 patients). Sixteen reactions (12.3%) in 10 patients were moderate and only 6 severe AEs (6.4%)were recorded in 4 patients (Supplementary Table S2). In terms of severity, no significant difference was observed by route, although the numbers were small for comparisons. Among 18 adverse reactions recorded according to Muller, there were only one grade 3 and one grade 4 reaction. Among the patients with severe AE, 3 were male and one female; all reported more than one reaction (one patient had 3 severe reactions); 3 were receiving SCIT vs. 1 SLIT; 3 reported AE during up‐dosing and one in maintenance; 2 were receiving AIT to grass, one to mites and one to parietaria; 3 had allergic rhinitis, while 1 had asthma and rhinitis). A detailed description of the severe cases is shown in Table 3.

**TABLE 3:** Characteristics of the patients with severe AE.

	Patient 1	Patient 2	Patient 3	Patient 4
Age (years old)	11	15	10	14
Sex	Female	Male	Male	Male
Route	SCIT	SLIT	SCIT	SCIT
Indication for AIT	Asthma and AR	AR	AR	AR
Baseline medication	SABA, ICS, NsCS	NsCS, Antihistamines	NsCS, Antihistamines	NsCS, Antihistamines
Number of AIT treatments	1	2	1	2
Number of AE	3	2	4	2
Severe AE	First, second and third reaction	second reaction	Fourth reaction	First reaction
Sensitization profile	Polysensitized	Polysensitized	Polysensitized	Polysensitized
Current AIT	Mites	Parietaria	Grass	Grass
Type of extract	Natural	Natural	Natural	Natural
Adjuvant	Aluminum hydroxide	‐	Tyrosine	Tyrosine
Schedule	Cluster	Conventional	Conventional	Conventional
Regimen	Perennial	Perennial	Perennial	Perennial
Phase of AE	Maintenance	Up‐dosing	Up‐dosing	Up‐dosing
AE onset (in hours: minutes)	<30 min	2h and 30 min	4 min	25 min
AE resolution (in hours: minutes)	>1 h	1h	1 h	40 min
Cofactors	None	Gastric ulcer	None	None

Abbreviations: AIT, allergen immunotherapy; AE, adverse events; ICS, inhalator corticosteroids; NsCS, nasal corticosteroids; SABA, short acting beta agonist.

AEs were recorded mostly during build‐up and early maintenance. Most AEs were reported during up‐dosing (52.7%) or early (4–6 weeks) maintenance (45.7%) compared to later maintenance (>6 weeks) (1.6%). The frequency of AE was marginally higher during the build‐up compared to early maintenance for both SCIT and SLIT treatments (*p* = 0.051). There was no difference regarding severity between build‐up and early maintenance; however, no severe reactions were observed after 6 weeks of maintenance. Two reactions observed in later maintenance were associated with SLIT treatments and were mild/moderate (Table 4).

**TABLE 4:** Adverse events phase according to route and severity.

	Total	Up‐dosing	Maintenance (4–6 weeks)	Maintenance (>6 weeks)
Severity of AE				
Mild	107 (83%)	59 (55%)	47 (44%)	1 (0.9%)
Moderate	16 (12.4%)	6 (37.5%)	9 (56.3%)	1 (6.3%)
Severe	6 (4.6%)	3 (50%)	3 (50%)	0
Route*				
SCIT	104 (80.6%)	55 (53%)	49 (47%)	0
SLIT	25 (19.4%)	13 (52%)	10 (40%)	2 (8%)
Total	**129 (100%)**	**68 (52.7%)**	**59 (45.7%)**	**2(100%)**

*Note*: Only AE for which severity was recorded are included. The frequency of AE was marginally higher during up‐dosing compared to early maintenance for both SCIT and SLIT. Bold values indicate the total number of adverse events according to the phase of reactions.

*p = 0.051.

**FIGURE 1:**
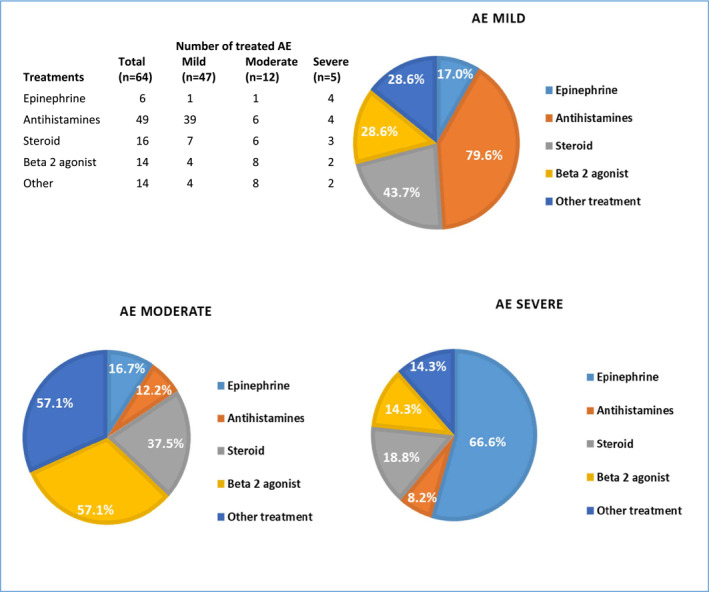
Treatment of AE according to reaction severity. Epinephrine was administered in 6 AEs (4/6 severe). Antihistamines were used more frequently in mild AE and Beta 2 agonists in moderate AE.

Epinephrine was used on only 6 occasions (4/6 were severe) (*p* < 0.001). Oral antihistamines were used more frequently to treat AE (76.5% of cases), followed by steroids in 25% and beta‐2 agonists in 21.9% of reactions (Figure 1). Modification of the AIT treatment was decided following 24 reactions that occurred in 12 patients (1.4%). Discontinuation of AIT was decided for 4 patients (0.4%), following 4 reactions (one severe and 3 moderate), lowering of dose after 12 AE, change in the build‐up schedule in 6 and change in the formulation in 2 AE. No fatalities were recorded.

### 3.3 Time interval of onset and duration of AE

The elapsed time from last AIT administration to AE onset and resolution was recorded in 104 reactions. The mean time of onset was 27 min, with a minimum of 4 min and a maximum of 5 h. The mean duration of AEs was 1.5 h (ranging from 5 min to nearly 24 h). The time of AE onset differed significantly between SCIT and SLIT (AE occurring during SCIT had an earlier onset compared to SLIT) (*p* = 0.013); there was however no difference in the mean duration (Supplementary Table S2).

The onset time was also grouped into three categories: a) < 30 min, b) 30–60 min, and c) > 60 min, to evaluate the relationship with the severity of systemic reactions (SR). Most SR (87%) occurred less than 30 min after AIT administration. Among severe SR, 4 out of 6 had an onset of <30 min while 2 reactions had a later onset. Most of these early onset SR (81%) were mild. Only 4/96 (4.2%) SR had an onset of >60 min; however, these included 1 mild, 2 moderate and 1 severe SR (Table 5).

**TABLE 5:** Onset time of systemic adverse events in relation to severity.

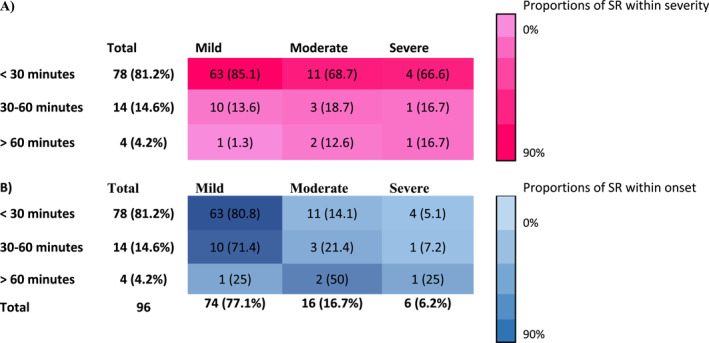

*Note*: Systemic adverse events (for those with available data) according to onset time and severity are presented by A) proportions within severity and B) proportions within onset. Bold values indicate the total number of SR by rows (onset) and columns (severity).

Abbreviation: SR, systemic reactions.

### 3.4 Risk factors for adverse events

Univariate analysis was performed for the following variables: age, sex, allergic condition, comorbidities, number of treatments, sensitization profile, previous AIT, current AIT, adjuvant and type of allergen for SCIT only to assess risk factors for AE (Supplementary Table S3). Patients sensitized to epithelia were significantly more likely to experience AE (odds ratio (OR) for [95% confidence interval (CI)] = 1.751 [1.017–3.015], *p* = 0.043). The subcutaneous route was associated with a significantly higher risk for AE compared to the sublingual route (OR [95% CI]) = 3.592 [2.164–5.963], *p* < 0.001). Age (OR [95% CI] = 1.057 [0.992–1.126], *p* = 0.085), patients with food allergy (OR [95% CI] = 2.448 [0.945–6.337], *p* = 0.065) and those receiving AIT with grass pollen (OR [95% CI] = 1.641 [0.985–2.735], *p* = 0.057) were marginally associated with higher odds for AE. In contrast, AIT with mites was marginally less likely associated with the occurrence of AE (OR [95% CI] = 0.653 [0.405–1.052], *p* = 0.080). In the separate univariate analysis for SCIT treatments, calcium phosphate was marginally less likely associated with AE compared with tyrosine (OR [95% CI] = 0.381 [0.138–1.050], *p* = 0.062) (Supplementary Table S3). The multivariate logistic regression including the above‐mentioned variables showed that the subcutaneous route was the only significant risk factor for AE (OR [95% CI]) = 4.336 [2.523–7.450], *p* = 0.001) (Supplementary Table S4).

## 4 DISCUSSION

This is the first report on the real‐life safety of AIT for respiratory allergy from a multinational registry. Adverse events due to AIT in children have been mostly assessed in either the context of clinical trials or surveys.^9^, ^10^, ^23^, ^24^ The EAACI first international retrospective survey recently evaluated AIT practical aspects and safety during the COVID‐19 pandemic, generating important real world data. The report showed no concerns regarding AIT tolerability.^33^ The role of registries as a robust, real‐world data gathering tool has been highlighted recently.^11^ BRIT, a UK‐based prospective registry, has reported outcomes from the UK National Health System on the efficacy and safety of AIT and other immunomodulatory treatments for chronic spontaneous urticaria, reflecting real‐life clinical practice.^12^, ^13^ In contrast, ADER encompasses a wider picture, mirroring how AIT works in 8 different centers in real life with all the expected heterogeneity, making this registry a valuable additional resource of real world evidence. Considering that ADER is focused on AIT safety, our results cover the full spectrum of AE characteristics and describe in detail all AE occurred during AIT treatment. Moreover, the dataset offers the possibility to assess factors that can lead to AE.

Our results are reassuring in that both SCIT and SLIT treatments are well tolerated in children and adolescents. AEs were recorded in overall about 10% of patients. The range of local and systemic reactions reported from previous studies is as low as 1.5%^23^ up to 17.9%.^25^, ^26^, ^27^, ^28^ This is not surprising, taking into account that patient selection, type of product, adjuvant and route of administration as well as intensity of follow‐up may affect safety outcomes and vary considerably. ADER is a multicenter registry that includes patients from 8 countries with different characteristics, a wide spectrum of AIT products and administration protocols. In fact, within ADER, the range of AE ratios between countries was very wide, although the higher proportions were observed in countries with small sample sizes.

Unsurprisingly, significantly less AEs were observed with SLIT than with SCIT.^5^ In fact, up to 90% of all AEs occurred during SCIT, while the ratio of patients suffering from an AE was more than 3 times lower in SLIT (4.9% vs. 15.5%). Nevertheless, in all cases, we confirmed that the vast majority of reactions in both SCIT and SLIT were mild.

In the univariate analysis, SCIT with grass pollen and epithelia had a higher risk for AE compared with other allergen sources. However, these associations were lost in the multivariate analysis, in which only the route of administration remained significant. Grass extract has also been associated with a higher rate of AE in other reports too.^10^, ^23^, ^26^ Many studies have assessed risk factors associated with AE^7^, ^8^, ^9^, ^10^, ^23^, ^26^ highlighting uncontrolled asthma, previous SR, or administration errors as the most frequently reported risk factors. In our population, asthma was not identified as a risk factor for severe AE; however, respiratory symptoms were one of the prevailing presentations of SR.

SR occurred predominantly (>80%) within 30 min from the administration, reinforcing recommendations on a standard 30 min observation period.^29^ However, we observed 4/96 reactions more than 60 min after administration, 3 of which were moderate to severe. This underpins the need for an individual risk assessment as well as potentially expanded safety precautions.^30^

Of importance, severe reactions were rare: 6 in about 1000 AIT courses (0.47% of patients) and occurred mostly during SCIT. Nevertheless, there was one severe reaction in a patient receiving SLIT drops. The reaction had a late onset, about 2 h after administration, and occurred during the build‐up. Active gastric ulcer was an apparently important cofactor in this patient; rapid uptake and/or minimal gastric acid cleavage of the allergen may have led to such a reaction. The reported incidence of severe reactions related to SLIT in the literature is very low (one case per 100 million administrations).^31^ However, it has been highlighted that they might be underreported due to the fact that most of them occur outside medical settings and are self‐reported.^32^ The data from Eudra Vigilance (European database of suspected adverse drug reactions from Europe) reported 82 cases of suspected anaphylaxis to house dust mite SLIT products from 2016‐2019.^34^ Considering the above, the need for educating patients on early recognition of adverse reactions occurring outside the doctor's office remains. A careful and individual evaluation of patients for possible cofactors that can lead to AE is an important step before prescribing AIT that should not be neglected by physicians.

An important observation from our data is the exceedingly rare occurrence of AE during late (>6 weeks) maintenance. In fact, AE during build‐up and early maintenance occurred with a comparable frequency. However, no severe reactions were observed in the late maintenance phase. It is possible that this corresponds to a time window for key immunological changes. Independently, while this observation needs further exploration, it suggests ‘extending’ the high alertness build‐up phase to 6 weeks. Dividing maintenance in an early and late phase in future studies can be helpful in this regard.

Even though more than 50% of the patients were polysensitized, the majority (90%) received single allergen immunotherapy, reflecting the ‘European’ approach to AIT, in contrast to the US approach of prescribing mixtures.^30^ Although there was no difference in the rate of AE in our population between those receiving mixtures or single allergen, it is still controversial whether treatment with mixtures can be approximately safe.^35^, ^36^

We found no significant differences between natural and allergoid preparations or in regard to different adjuvants, probably reflecting the small number of overall AEs, and possibly, the fact that our population consisted of children and adolescents. The adult population will be analyzed separately in the forthcoming papers.

In spite of the 250 recorded AE, discontinuation of immunotherapy was decided in only 4 patients (0.46%), further supporting the finding that the treatment was well‐tolerated and AEs were perceived as mild from both parents and the children themselves but also by physicians.

The current report has some limitations. The variability between centers, although a strength, can also be a limitation when comparing different small subsamples. For example, the proportions of SCIT and SLIT, but also different preparations vary considerably in our registry. SLIT follow‐up is usually different from SCIT. Inclusion criteria were not identical as each country has its own health system and regulations. However, having included different clinical settings, patient populations, physician's educational background and AIT protocols and products, according to real‐life clinical practice, empowers this study findings and offers a large amount of real‐world evidence.

AIT remains a key feature of personalized medicine for allergic disease treatment, continuously evolving and comprises a major part of an allergist's daily practice worldwide. Considering that it is crucial to shape future recommendations based on broad applicable and credible data, such as those from registries with a prospective approach.^37^ The design and setup of ADER allows data entering in a simple and effective manner that permits easy expansion in other countries. In addition, we recorded SRs using the harmonized MeDRA terminology^19^ as in the EAASI, which is a strength. The use of MeDRA terminology, recommended also by EMA, offers a uniform system of collecting safety data that enable comparing results from different reports and limits recording biases.

In conclusion, this study demonstrates the safety profile of SCIT and SLIT treatments in children and adolescents. While SLIT results in significantly less AE than SCIT, both approaches are safe and well tolerated in specialized centers across different countries.

## AUTHOR CONTRIBUTIONS

**Julijana Asllani**: data curation (equal); formal analysis (equal); writing original draft (lead). **Dimitrios Mitsias**: project administration (lead), methodology (equal), data curation (equal), and formal analysis (equal). **George Kostantinou**: data curation (equal); formal analysis (equal). **Todor Popov**: conceptualization (equal). **Nikos G. Papadopoulos**: conceptualization: (equal); methodology (equal), funding acquisition (lead). **Moises Calderon**: conceptualization (equal); methodology (equal). **All authors**: Investigation (equal); writing‐review and editing (equal).

## CONFLICT OF INTEREST STATEMENT

Nikos G. Papadopoulos has received grants from Capricare, Nestle, Numil, Vianex and/or fees for consultancy from Abbott, Abbvie, Astra Zeneca, GSK, HAL, Medscape, Menarini/Faes Farma, Mylan, Novartis, Nutricia, OM Pharma, Regeneron/Sanofi in the last 3 years. Dimitrios Mitsias is or recently was a speaker for AstraZeneca, Gerolymatos International, Novartis, Sanofi, in the last 3 years. GNK is or recently was a speaker and/or advisor for and/or has received research funding from AstraZeneca, Chiesi, GSK, Menarini, Novartis, Pfizer, Sanofi, and Vianex in the last 3 years. The other authors have no conflicts of interest to declare in relation to this work.

## Supporting information

Supporting Information S1
