# Supplementary material for: Adverse events in children and adolescents undergoing allergen immunotherapy for respiratory allergies—Report from the Allergen Immunotherapy Adverse Events Registry (ADER), a European Academy of Allergy and Clinical Immunology taskforce
Source: Clin Transl Allergy. 2023 Jun 2;13(6):e12250. doi: 10.1002/clt2.12250 (PMC10236500; doi:10.1002/clt2.12250)
Supplement: Supplementary file 1 — Supporting Information S1 [file CLT2-13-e12250-s001.docx]

# Online Supplement

**Supplementary Table 1. Adverse event symptoms recorded in patients receiving SCIT and SLIT (according to MeDRA classification for adverse reactions).**

| **Symptoms** | **Total**  **n (%)** | **SCIT** | **SLIT** |
| --- | --- | --- | --- |
| Abdominal pain | 2 (2) | 2 | 0 |
| Angioedema | 2 (2) | 1 | 1 |
| Asthma | 3 (3) | 3 | 0 |
| Hypotension | 1 (1) | 1 | 0 |
| Bronchospasm | 7 (7) | 6 | 1 |
| Chest discomfort | 4 (4) | 3 | 1 |
| Chest Tightness | 9 (9) | 8 | 1 |
| Conjunctivitis | 5 (5) | 4 | 1 |
| Cough | 48 (48) | 46 | 2 |
| Diarrhea | 1 (1) | 1 | 0 |
| Dyspnea | 11 (11) | 9 | 2 |
| Dizziness | 1 (1) | 1 | 0 |
| Local erythema | 4 (4) | 3 | 1 |
| Flushing | 3 (3) | 3 | 0 |
| Generalized erythema | 1 (1) | 1 | 0 |
| Headache | 7 (7) | 6 | 1 |
| Hypotension | 1 (1) | 1 | 0 |
| Pruritus | 6 (6) | 3 | 3 |
| Rhinitis | 43 (43) | 38 | 5 |
| Foreign body | 1 (1) | 0 | 1 |
| Localized urticarial | 10 (10) | 6 | 4 |
| Generalized urticarial | 7 (7) | 6 | 1 |
| Vomiting | 2 (2) | 2 | 0 |
| Wheezing | 3 (3) | 2 | 1 |

Symptoms such as: dysphonia, dysphagia, fatigue, loss of consciousness and syncope were not reported and thus not included.

**Supplementary Table 2. Time of onset and severity of AE by route of administration**

|  | | **Total AE** | **AE during SCIT**  **n (%)** | **AE during SLIT**  **n (%)** |
| --- | --- | --- | --- | --- |
| **Number of adverse events** | | **250** | **225 (90)** | **25 (10)** |
| Mean time of onset and range of AE (in hours: minutes)* | | 00:27 (00:04-05:00) | 00:23 | 00:41 |
| Mean time of duration and range of AE (in hours: minutes) | | 01:33 (00:05-24:00) | 1:36 | 1:22 |
| Muller Grading severity † | |  |  |  |
|  | Grade I | 8 (3.2) | 6 (75) | 2 (25) |
|  | Grade II | 8 (3.2) | 6 (75) | 2 (25) |
|  | Grade III | 1 (0.4) | 1 (100) | 0 (0) |
|  | Grade IV | 1 (0.4) | 1 (100) | 0 (0) |
| *Severity of AE* † | | 130 | 105 | 25 |
|  | Mild | 108 (83.1) | 87 (80.5) | 21 (19.4) |
|  | Moderate | 16 (12.3) | 13 (81.2) | 3 (18.7) |
|  | Severe | 6 (4.6) | 5 (83.3) | 1(16.6) |
| *Local reactions* | | 89 (35.6) | 75 (30) | 14 (5.6) |
|  | Mild oral or skin symptoms | 59 (23.6) | 50 (84.7) | 9 (15.2) |
|  | Large local reactions | 25 (10) | 25 (100) | 0 (0) |
|  | Oral edema | 5 (2) | 0 (0) | 5 (100) |

*Statistically significant differences between SCIT and SLIT for the specific variables (p<0.005). †AE for which severity was recorded are presented.

**Supplementary Table 3. Univariate analysis to evaluate risk factors in patients with and without AE.**

| **Factors related with patient adjusted for age, sex and route.** | **OR** | **p** | **Lower CI 95% level** | **Upper CI 95% level** |
| --- | --- | --- | --- | --- |
| Sex (Male VS female) | 0.883 | 0.601 | 0.553 | 1.410 |
| Age (continuous) | 1.057 | *0.085* | 0.992 | 1.126 |
| Age (<11 y old vs.>12 y old) | 0.532 | 0.165 | 0.218 | 1.296 |
| Atopic dermatitis (yes vs. no) | 1.138 | 0.749 | 0.516 | 2.510 |
| Food allergy (yes vs. no) | 2.448 | *0.065* | 0.945 | 6.337 |
| Asthma vs. AR only | 1.286 | 0.313 | 0.789 | 2.094 |
| Asthma vs. AR and asthma | 0.888 | 0.815 | 0.330 | 2.394 |
| Previous AIT (yes vs. no) | 1.667 | 0.238 | 0.713 | 3.895 |
| Sensitization to pollen | 0.783 | 0.311 | 0.487 | 1.258 |
| Sensitization to mites | 0.719 | 0.165 | 0.451 | 1.146 |
| Sensitization to molds | 0.656 | 0.217 | 0.336 | 1.281 |
| Sensitization to epithelia | 1.751 | **0.043*** | 1.017 | 3.015 |
| Polysensitized vs. Monosensitized | 0.856 | 0.512 | 0.539 | 1.361 |
| Receiving two AIT treatments vs. one AIT treatment | 0.964 | 0.910 | 0.507 | 1.831 |
| AIT with grass | 1.641 | *0.057* Ɨ | 0.985 | 2.735 |
| AIT with mites | 0.653 | *0.080* ‡ | 0.405 | 1.052 |
| AIT with epithelia | 1.917 | 0.270 | 0.603 | 6.092 |
| AIT with molds | 0.425 | 0.109 | 0.149 | 1.210 |
| AIT with olive | 1.572 | 0.330 | 0.633 | 3.904 |
| AIT with parietaria | 1.517 | 0.375 | 0.604 | 3.809 |
| SCIT versus SLIT | 3.592 | **0.000**** | 2.164 | 5.963 |
| **SCIT treatments only** |  |  |  |  |
| Natural vs. allergoid | 0.755 | 0.674 | 0.204 | 2.798 |
| Aluminum vs. tyrosine | 0.576 | 0.196 | 0.249 | 1.329 |
| *Calcium vs. tyrosine* | *0.381* | *0.062* § | *0.138* | *1.050* |

*Statistically significant differences (p<0.05). ** p<0.001. Odds ratio (OR) for 95% Confidence Interval (CI) were calculated. Ɨ AIT with grass extract was marginally associated with a higher risk for AE. ‡ AIT with mites was marginally less likely for adverse reactions. § SCIT treatments with calcium phosphate as an adjuvant was marginally less likely associated with AE compared to tyrosine. Abbreviations: SCIT-subcutaneous, SLIT- sublingual, AIT-allergen immunotherapy, AR-allergic rhinitis.

**Supplementary Table 4. Evaluation of risk factors using a multivariate logistic regression analysis.**

| **Variables** | **P** | **OR** | **95% CI. For OR** | |
| --- | --- | --- | --- | --- |
|  |  |  | **Lower** | **Upper** |
| Age | 0.344 | 1.033 | 0.966 | 1.105 |
| Gender (males vs. females) | 0.404 | 0.811 | 0.496 | 1.326 |
| SCIT vs. SLIT | **0.000*** | 4.388 | 2.546 | 7.563 |
| Polysensitized vs. Monosensitized | 0.788 | 1.089 | 0.586 | 2.024 |
| Atopic dermatitis (yes vs. no) | 0.836 | 0.912 | 0.379 | 2.195 |
| Food allergy (yes vs. no) | 0.111 | 2.419 | 0.817 | 7.162 |
| Sensitization to pollen | 0.736 | 1.112 | 0.601 | 2.058 |
| Sensitization to mites | 0.872 | 0.940 | 0.445 | 1.988 |
| Sensitization to epithelia | 0.058 | 1.903 | 0.978 | 3.700 |
| Sensitization to molds | 0.367 | 0.659 | 0.266 | 1.631 |
| Previous AIT (yes vs. no) | 0.376 | 1.500 | 0.612 | 3.677 |
| AIT with grass | 0.691 | 1.226 | 0.448 | 3.355 |
| AIT with olive | 0.895 | 1.079 | 0.348 | 3.341 |
| AIT with parietaria | 0.887 | 1.089 | 0.336 | 3.523 |
| AIT with alternaria | 0.398 | 0.478 | 0.087 | 2.644 |
| AIT with mites | 0.698 | 0.778 | 0.219 | 2.761 |
| AIT with epithelia | 0.962 | 0.962 | 0.190 | 4.855 |
| Asthma vs. AR only | 0.663 | 0.799 | 0.292 | 2.191 |
| AR and asthma vs. AR only | 0.315 | 0.766 | 0.455 | 1.289 |

*Statistically significant difference (p<0.001). SCIT was significantly associated with a higher risk for AE compared to SLIT. Odds ratio (OR) for 95% Confidence Interval (CI) were calculated for each variable. Abbreviations: SCIT-subcutaneous, SLIT- sublingual, AIT-allergen immunotherapy, AR-allergic rhinitis, OR-odds ratio, CI- confidence Interval.
